# A double-negative feedback loop between E2F3b and miR- 200b regulates docetaxel chemosensitivity of human lung adenocarcinoma cells

**DOI:** 10.18632/oncotarget.8376

**Published:** 2016-03-25

**Authors:** Yanping Gao, Longbang Chen, Haizhu Song, Yitian Chen, Rui Wang, Bing Feng

**Affiliations:** ^1^ Department of Medical Oncology, Jinling Hospital, School of Medicine, Nanjing University, Nanjing 210002, China

**Keywords:** lung adenocarcinoma (LAD), chemoresistance, miR-200b, E2F3b, feedback loop

## Abstract

MicroRNAs (miRNAs) are non-coding small RNAs which negatively regulate gene expressions mainly through 3′-untranslated region (3′-UTR) binding of target mRNAs. Recent studies have highlighted the feedback loops between miRNAs and their target genes in physiological and pathological processes including chemoresistance of cancers. Our previous study identified miR-200b/E2F3 axis as a chemosensitivity restorer of human lung adenocarcinoma (LAD) cells. Moreover, E2F3b was bioinformatically proved to be a potential transcriptional regulator of pre-miR-200b gene promoter. The existance of this double-negative feedback minicircuitry comprising E2F3b and miR-200b was confirmed by chromatin immunoprecipitation (ChIP) assay, site-specific mutation and luciferase reporter assay. And the underlying regulatory mechanisms of this feedback loop on docetaxel resistance of LAD cells were further investigated by applying *in vitro* chemosensitivity assay, colony formation assay, flow cytometric analysis of cell cycle and apoptosis, as well as mice xenograft model. In conclusion, our results suggest that the double-negative feedback loop between E2F3b and miR-200b regulates docetaxel chemosensitivity of human LAD cells mainly through cell proliferation, cell cycle distribution and apoptosis.

## INTRODUCTION

Lung cancer is one of the most common malignancies and the leading cause of cancer-related deaths worldwide [1]. Approximately 70%-80% of lung cancers are non-small cell lung cancer (NSCLC), including adenocarcinoma, squamous cell carcinoma, and large cell carcinoma. Despite the advances in diagnostic and therapeutic techniques, most patients with lung cancer are already at advanced stage when diagnosed, and the overall 5-year survival rate is still poor [2]. Chemoresistance remains one of the major obstacles restricting the clinical application of anti-cancer drugs, and the possible mechanisms include both genetic and epigenetic dysregulations of key genes and proteins involved in drug transportion and metabolism, cell cycle distribution, apoptosis and autophagy [3–5]. MicroRNAs (miRNAs) are endogenous small non-coding RNAs, 19-22 nucleotides in length that regulate about 30% of human gene expression [6]. MiRNAs regulate gene expression post-transcriptionally mainly through 3′-untranslated region (3′-UTR) binding of target mRNAs, causing either degradation or inhibition of gene translation [7, 8], thereby playing regulatory roles in complex physiological and pathological processes such as cell differentiation, proliferation, apoptosis and tumorigenesis [9, 10]. Emerging evidence indicates that miRNAs could act as both oncogenes and tumor-suppressor genes in cancers [11–17]. Dysregulation of specific miRNAs contributes to carcinogenesis and chemoresistance of human malignancies including lung cancer [18, 19].

Based on our previous miRNA microarray data, miR-200b was identified as the most down-regulated miRNA in docetaxel-resistant human lung adenocarcinoma (LAD) SPC-A1/DTX cells compared with parental SPC-A1 cells [20]. Restoration of miR-200b could boost *in vitro* and *in vivo* chemosensitivity of LAD cells by, at least partially, post-transcriptional down-regulation of E2F3, which was critical for the maintenance of normal cell cycle progression [21]. Moreover, E2F3 was bioinformatically identified as a potential transcriptional regulator of pre-miR-200b gene promoter, suggesting a double-negative feedback minicircuitry comprising E2F3b and miR-200b. The results of the present study confirmed the existance of this feedback loop and showed, for the first time, that the double-negative feedback loop between E2F3b and miR-200b could regulate docetaxel chemosensitivity of human LAD cells mainly through cell proliferation, cell cycle distribution and apoptosis.

## RESULTS

### Bioinformatical identification of the direct binding of E2F3 upon miR-200b gene

**Figure 1:**
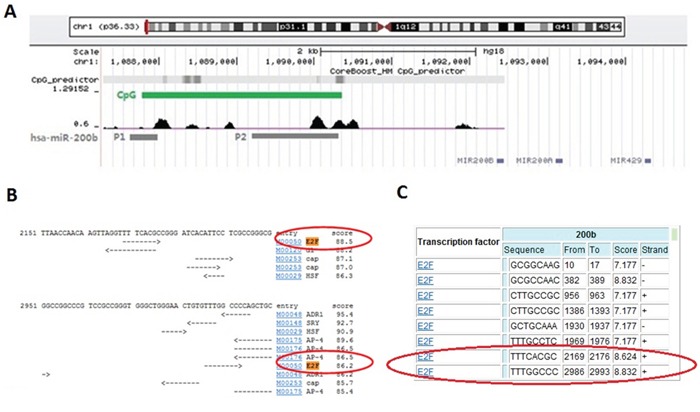
Bioinformatical evidence of the direct binding of E2F3 upon miR-200b gene **A.** CoreBoost_HM (http://rulai.cshl.edu/tools/CoreBoost_HM/) on-line analysis was used to identify the promoter regions of miR-200b (named as P1 and P2). **B.** TFSEARCH (http://www.cbrc.jp/research/db/TFSEARCH.html) and **C.** CONSITE (http://asp.ii.uib.no:8090/cgi-bin/CONSITE/consite) on-line softwares were performed to find the potential E2F3 binding sites in miR-200b promoter.

By using the on-line miRNA gene promoter predictor CoreBoost_HM (http://rulai.cshl.edu/tools/CoreBoost_HM/), two separated promoters (P1 and P2) of miR-200b were identified 4.5 kb and 2 kb upstream the miR-200b gene, respectively (Figure 1A), which was in accordance with previous studies [22, 23]. By further applying the on-line transcription factor binding site analysis softwares TFSEARCH (http://www.cbrc.jp/research/db/TFSEARCH.html) and CONSITE (http://asp.ii.uib.no:8090/cgi-bin/CONSITE/consite), a potential binding site of E2F3 (5 '- TTTC[A] CGC - 3′) was identified upon the P2 promoter (Figure 1B and 1C).

### Functional identification of the direct binding of E2F3b upon miR-200b gene

**Figure 2:**
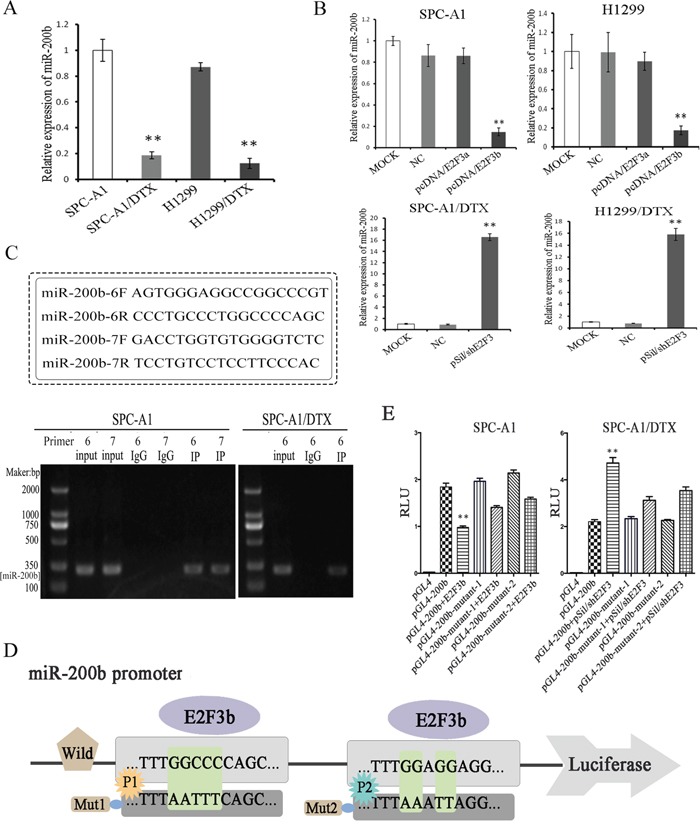
Functional evidence of the direct binding of E2F3b upon miR-200b gene Quantifications of miR-200b expression in docetaxel-resistant LAD cells (SPC-A1/DTX and H1299/DTX) and their parental cells (SPC-A1 and H1299) before **A.** and after **B.** manipulated E2F3a and E2F3b expressions were achieved by qRT-PCR. MiRNA abundance was normalized to U6 RNA. **C.** ChIP assays were performed in SPC-A1 and SPC-A1/DTX cells with antibodies against E2F3 or IgG control. **D.** Schematic representation of miR-200b gene promoter with the putative E2F3b-binding sites and the sequences of the point mutations. **E.** Luciferase reporter assays in SPC-A1 and SPC-A1/DTX cells with co-transfection of the pGL4 basic firefly luciferase reporters containing wild or mutated miR-200b promoter sequences and E2F3b or pSil/shE2F3 plasmid vectors as indicated. Data were normalized to luciferase activity and determined relative to empty vector promoter activity and presented as mean±SEM of three independent experiments each performed in triplicate. *p< 0.05, **p < 0.01 vs. control group.

Coincide with our previous study, the expression levels of miR-200b were enormously down-regulated in both SPC-A1/DTX and H1299/DTX cells in comparison with the parental SPC-A1 and H1299 cells, respectively (*p*<0.01) (Figure 2A). Subsequently, manipulation of E2F3a and E2F3b expression was achieved via plasmid vectors introduction. It was found that inhibition of E2F3 significantly elevated the levels of miR-200b in both SPC-A1/DTX and H1299/DTX cells compared with the control groups (*p*<0.01). Interestingly, miR-200b levels were notably decreased after E2F3b up-regulation in SPC-A1 and H1299 cells (*p*<0.01), while few changes were observed after E2F3a up-regulation (Figure 2B).

To determine whether E2F3 could directly interact with miR-200b promoter, chromatin immunoprecipitation (ChIP) assay was applied. 10 pairs of primers in total (named no.1∼10 primers) were designed using Primer5.0. In SPC-A1 cells, E2F3 regulation sites were located in no.6 and 7 primers corresponding areas within the promoter site of miR-200b, while in SPC-A1/DTX cells, E2F3 regulation site was only located in no.6 primer corresponding area (Figure 2C). Considering the diverse functions between the two cell lines, it was deduced that the no.6 primer corresponding area may be more conservative.

To further confirm the direct binding and function of E2F3b upon miR-200b, both wild and mutated miR-200b promoter sequences (towards P1 and P2, respectively) were designed and cloned into the pGL4 basic firefly luciferase reporters and co-transfected with E2F3b plasmid vectors into SPC-A1 and SPC-A1/DTX cells (Figure 2D). The augment of E2F3b significantly suppressed the luciferase activity of miR-200b luciferase promoter constructs (*p*<0.01). Moreover, when the binding sequences were mutated, the suppressive effects of E2F3b on luciferase activity were attenuated (Figure 2E), suggesting the direct negative regulation of E2F3b on the promoter region of miR-200b.

### E2F3b negatively regulates miR-200b expression and docetaxel chemosensitivity of LAD cells *in vitro*

**Figure 3:**
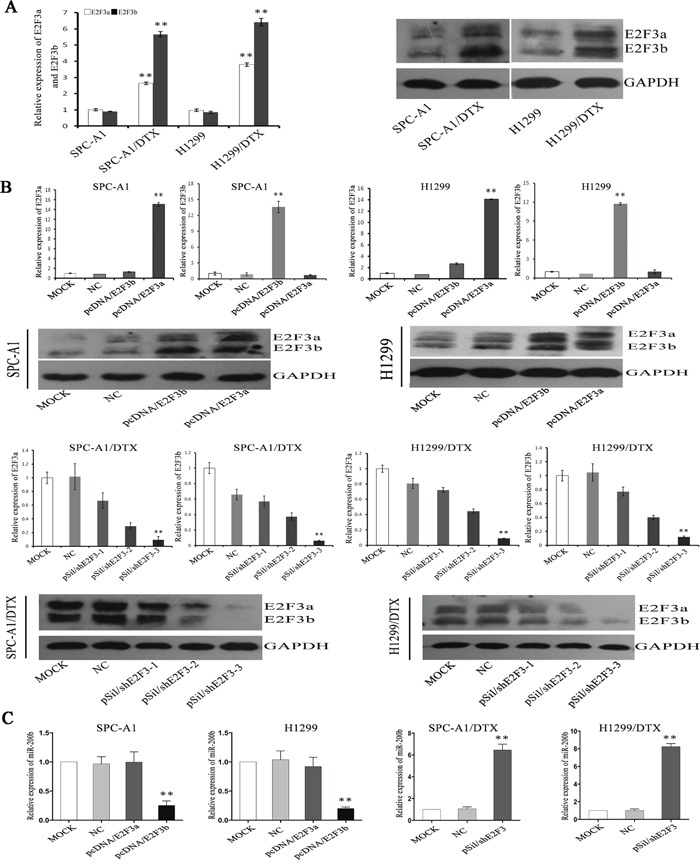
The regulation of E2F3a/b on miR-200b expression and chemosensitivity of LAD cells E2F3a/b mRNA and protein expression levels in SPCA1/DTX, H1299/DTX cells and the parental SPC-A1, H1299 cells before **A.** and after **B.** manipulated E2F3a and E2F3b expressions were detected by qRT-PCR and Western Blot analysis, respectively. Abundance of mRNA and protein was normalized to U6 RNA and GAPDH, respectively. **C.** Quantifications of miR-200b expression in different manipulated LAD cell lines as indicated was also achieved by qRT-PCR. Abundance of miRNA was normalized to U6 RNA. Data are representative of at least three independent experiments and are shown as mean±SEM. *p < 0.05, **p < 0.01 vs. control group.

Real-time quantitative RT-PCT (qRT-PCR) and Western Blot analysis showed that E2F3a and E2F3b levels were notably up-regulated at both mRNA and protein levels in docetaxel-resistant LAD cells compared with the parental cell lines (*p*<0.01) (Figure 3A). To determine whether E2F3a and/or E2F3b expression had an effect on the chemosensitivity of LAD cells, transfection of E2F3a, E2F3b eukaryotic expression vectors and E2F3 interference vector (named pcDNA/E2F3a, pcDNA/E2F3b, and pSil/shE2F3, respectively) was performed in LAD cell lines and satisfactory transfection efficiency was achieved at 48 hours after transfection (Figure 3B). In both SPC-A1 and H1299 cells, introduction of E2F3b, not E2F3a, resulted in a pronounced up-regulation of miR-200b expression (*p*<0.01). On the other hand, in both SPC-A1/DTX and H1299/DTX cells, miR-200b expression levels were significantly increased after artificial knockout of E2F3 gene (*p*<0.01) (Figure 3C).

**Figure 4:**
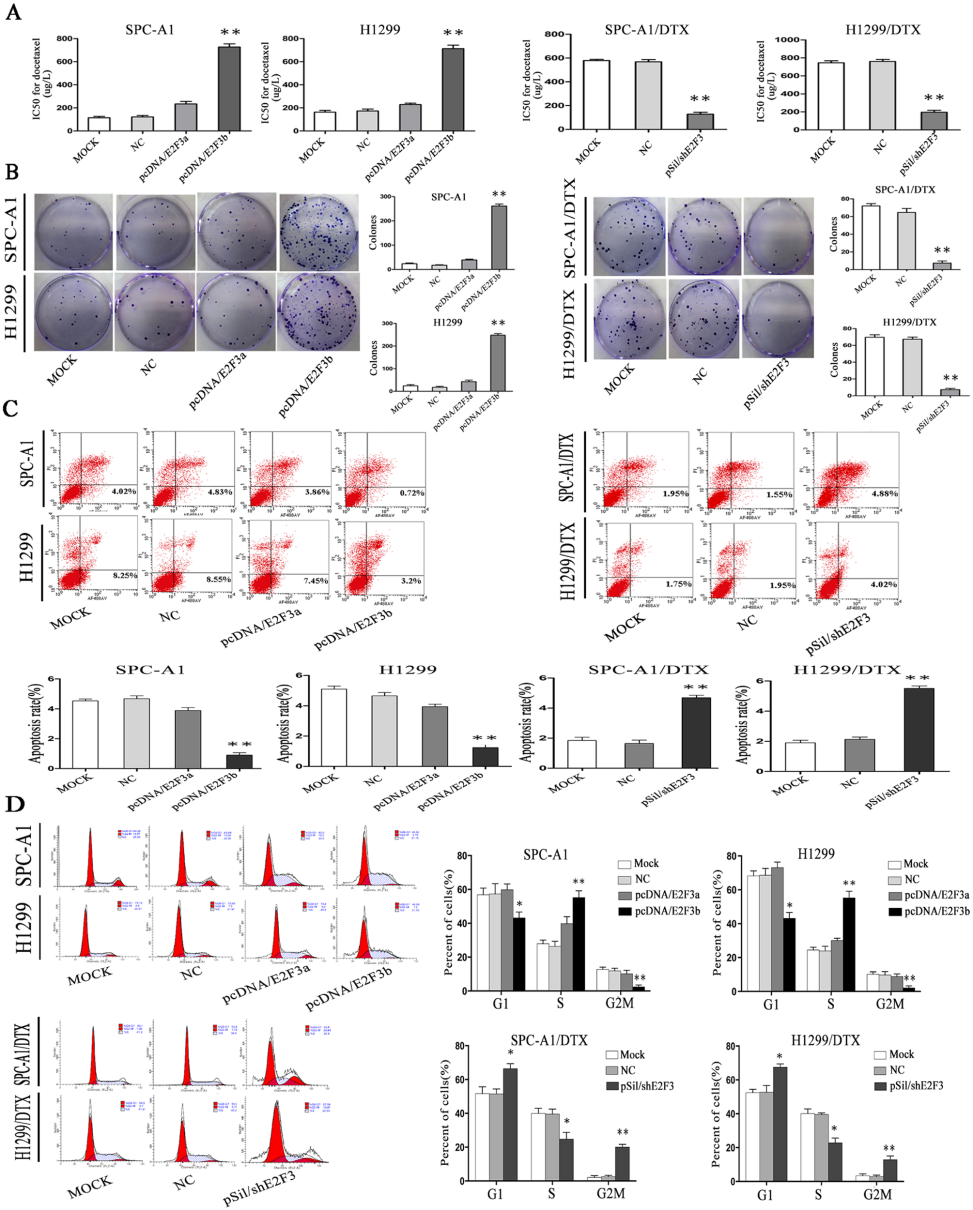
The *in vitro* effects of E2F3a/b on cell proliferation, apoptosis, cell cycle distribution, and response to docetaxel of LAD cells In SPCA1/DTX, H1299/DTX cells and the parental SPC-A1, H1299 cells, ectopic up- or down-regulation of E2F3a/b was achieved by transfection of pcDNA/E2F3a/b or pSil/shE2F3. **A.** IC50 values for docetaxel were measured by MTT assay. **B.** Cell proliferating ability was detected by colony formation assay. **C.** Cell apoptosis and **D.** cell cycle distribution data all came from flow cytometric analysis. Results are obtained in three independent experiments and are shown as mean±SEM. *p < 0.05, **p < 0.01 vs. control group.

Interestingly, after ectopic overexpression of E2F3b, the IC50 value for docetaxel significantly increased (*p*<0.01) (Figure 4A), indicating a potential regulatory role of E2F3b towards docetaxel chemosensitivity of LAD cells. In SPC-A1 cells, the IC50 value for docetaxel was 234.5±8.7μg/L and 727±11.2μg/L after E2F3a and E2F3b overexpression, respectively, in comparison with the NC group of 114±10.0μg/L. In H1299 cells, the IC50 value for docetaxel was 228.5±13.7μg/L and 713±15.1μg/L after E2F3a and E2F3b overexpression, respectively, in comparison with the NC group of 163±10.0μg/L. Moreover, in SPC-A1/DTX and H1299/DTX cells transfected with pSil/shE2F3, the IC50 values for docetaxel decreased from 568.5±11.2μg/L and 760.8±15.3μg/L to 127.4±16.2μg/L and 196.4±18.4μg/L, respectively. The above results suggested that E2F3b, rather than E2F3a, could negatively regulate miR-200b expression and docetaxel chemosensitivity of LAD cells through direct binding upon miR-200b gene.

### E2F3b affects cell proliferation, apoptosis, and cell cycle distribution of LAD cells *in vitro*

By applying colony formation assay, significantly enhanced proliferating ability was observed in SPC-A1 and H1299 cells transfected with E2F3b than that of E2F3a or NC-transfected cells (*p*<0.01), and significantly suppressed proliferating ability was observed in E2F3-knockout SPC-A1/DTX and H1299/DTX cells than that of NC-transfected cells (*p*<0.01) (Figure 4B), indicating the proliferative function of E2F3b *in vitro*.

To further analyze the mechanisms by which ectopic E2F3b expression promoted cell proliferation, flow cytometric analysis of apoptosis and cell cycle distribution were utilized. As shown in Figure 4C and 4D, enforced expression of E2F3b resulted in a dramatic decrease of apoptosis in SPC-A1 cells from 4.68±0.2% to 0.9±0.1% compared with the NC group (*p*<0.01), but also a decreased percentage of cells in G2/M phase from 11.8±2.9% to 2.4±0.8% (*p*<0.01) and an increased population in S phase from 26.4±3.0% to 55.5±4.2% (*p*<0.01). Vice versa, down-regulation of E2F3 resulted in an vivid enhancement of apoptosis in SPC-A1/DTX cells from 1.89±0.2% to 4.69±0.6% in comparison with the NC group (*p*<0.01), as well as a retardant in G2/M phase from 3.7±0.6% to 20.9±2.8% (*p*<0.01) and decreased population in S phase from 39.9±5.8% to 24.8±3.6% (*p*<0.05). Similar results were obtained in H1299 and H1299/DTX cells.

### E2F3b exerts *in vitro* functions in a miR-200b-dependent manner in LAD cells

**Figure 5:**
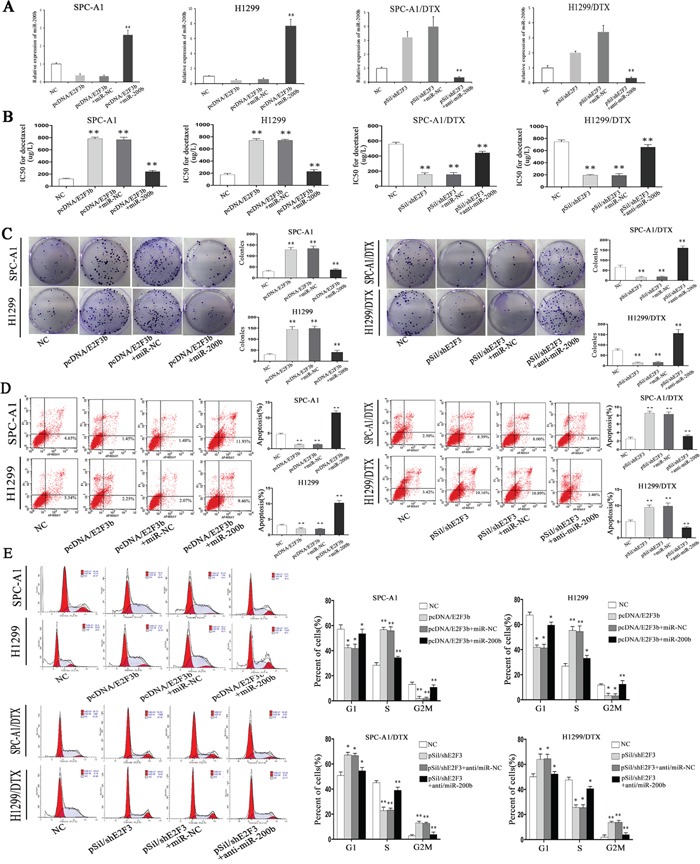
The role of miR-200b in the *in vitro* regulation of E2F3b on LAD cells PcDNA-NC, pcDNA/E2F3b vectors were transfected into SPC-A1 and H1299 cells without (or with) previous transfection of miR-200b mimics; sh-NC, pSil/shE2F3 vectors were transfected into SPC-A1/DTX and H1299/DTX cells without (or with) previous transfection of miR-200b inhibitors. **A.** Quantification of miR-200b expression was achieved by qRT-PCR. MiRNA abundance was normalized to U6 RNA. **B.** IC50 values for docetaxel were measured by MTT assay. **C.** Cell proliferating ability was detected by colony formation assay. **D.** Cell apoptosis and **E.** cell cycle distribution data all came from flow cytometric analysis. Error bars represent the mean±SEM of at least three independent experiments. *p < 0.05, **p < 0.01 vs. control group.

To determine whether E2F3b affected LAD cell proliferation, apoptosis, and cell cycle distribution in a miR-200b-dependent manner, rescue experiments were performed. In detail, pcDNA-NC, pcDNA/E2F3b vectors were transfected into SPC-A1 and H1299 cells without (or with) previous transfection of miR-200b mimics; sh-NC, pSil/shE2F3 vectors were transfected into SPC-A1/DTX and H1299/DTX cells without (or with) previous transfection of miR-200b inhibitors. QRT-PCR results indicated that the negative regulation of E2F3b upon miR-200b in SPC-A1 and H1299 cells could be abolished by co-transfection of miR-200b mimics, vice versa in SPC-A1/DTX and H1299/DTX cells (*p*<0.01) (Figure 5A). Intriguingly, the increasing effects of E2F3b on the IC50 values for docetaxel could be partially abrogated by co-transfection of miR-200b mimics in SPC-A1 and H1299 cells (*p*<0.01), vice versa in SPC-A1/DTX and H1299/DTX cells (*p*<0.01) (Figure 5B). Similarly, the increased colony formation capacity of SPC-A1 and H1299 cells owing to overexpression of E2F3b could be decayed after miR-200b mimics introduction. In SPC-A1/DTX and H1299/ DTX cells, down-regulation of E2F3b significantly lessened the colony formation amount, and the effect could also be partially reversed by miR-200b inhibitor introduction (*p*<0.01) (Figure 5C). Subsequently, flow cytometric assays indicated that the anti-apoptotic effect of E2F3b could be significantly reversed by miR-200b introduction in SPC-A1 and H1299 cells. This anti-apoptotic effect was also observed after co-transfection of pSil/shE2F3and miR-200b inhibitors in docetaxel-resistant LAD cells (*p*<0.01) (Figure 5D). Cell cycle analysis showed that up-regulation of E2F3b dramatically decreased the percentage of cells in G2/M phase in SPC-A1 and H1299 cells, which could be partially reversed by miR-200b introduction (*p*<0.01), vice versa in SPC-A1/DTX and H1299/DTX cells (*p*<0.01) (Figure 5E). It was therefore concluded that E2F3b affected cell proliferation, apoptosis, cell cycle distribution, and docetaxel chemosensitivity of LAD cells *in vitro*, at least partially, in a miR-200b-dependent manner.

### E2F3b negatively regulates miR-200b expression and docetaxel chemosensitivity of LAD cells *in vivo*

**Figure 6:**
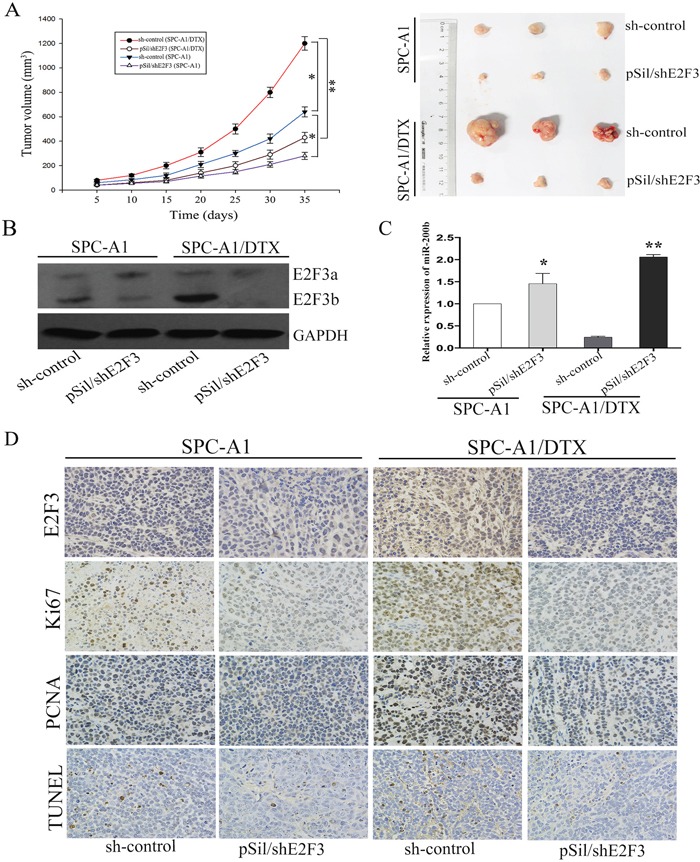
The *in vivo* effects of E2F3b on miR-200b expression and chemosensitivity of LAD cells SPC-A1 and SPC-A1/DTX cells were transfected with pSil/shE2F3 or pSil/shE2F3-NC and injected subcutaneously into nude mice. When the average tumor size reached about 50 mm^3^, docetaxel was given through intraperitoneal injection with a dose of 1 mg/kg, one dose every other day with 3 doses in total. **A.** Growth curve of tumor volumes and representative photographs of tumors formed 35 days after the first administration of docetaxel. **B.** Quantification of E2F3a/b protein in the transplanted tumor tissues by Western Blot analysis. **C.** Quantification of miR-200b in the transplanted tumor tissues by qRT-PCR. Abundance of protein and miRNA was normalized to GAPDH and U6 RNA, respectively. **D.** Immunostaining of E2F3, Ki-67 and PCNA protein and TUNEL assay stained sections of the transplanted tumors as indicated (original magnification, ×400). *p < 0.05, **p < 0.01 vs. control group.

To better understand the biological mechanisms underlying E2F3b-related docetaxel resistance of LAD cells *in vivo*, pSil/shE2F3 and pSil/shE2F3-NC–transfected SPC-A1 and SPC-A1/DTX cells were subcutaneously inoculated into nude mice. Almost 10 days after inoculation, all mice developed tumors. Docetaxel was applied via intraperitoneal injection at a dose of 1 mg/kg when the average tumor size reached about 50mm^3^, 1 dose every other day, with 3 doses in total [24]. As shown in Figure 6A, tumors derived from pSil/shE2F3 transfected SPC-A1 and SPC-A1/DTX cells grew substantially more slowly in comparison with the control ones, respectively. All mice were sacrificed 35 days after the first administration of docetaxel. The average size of pSil/shE2F3 transfected-SPC-A1 and SPC-A1/DTX cell-derived tumors were distinctly smaller than that of the control groups, respectively. Western Blot analysis indicated that the protein levels of E2F3b were significantly down-regulated in pSil/shE2F3 groups (Figure 6B). In addition, miR-200b expression levels were remarkably up-regulated in pSil/shE2F3 groups compared with the control groups in both SPC-A1 (*p*<0.05) and SPC-A1/DTX cells (*p*<0.01) via qRT-PCR (Figure 6C). Furthermore, immunostaining analysis and TUNEL staining revealed a lower positive rate of proliferating cell nuclear antigen (PCNA) and Ki67 as well as a higher apoptotic rate of tumors formed from pSil/shE2F3 transfected LAD cells in comparison with the control groups (Figure 6D), suggesting the critical role of E2F3b/miR-200b axis in modulating chemosensitivity of docetaxel-resistant LAD cells *in vivo*.

## DISCUSSION

The miRNA 200 family members, consisting of miR-200b, miR-200c, miR-429, miR-200a and miR-141, were considered to be tumor suppressor miRNAs in cancer development and progression [25, 26]. Among them, miR-200b was validated as a chemosensitivity restorer in cancers including NSCLC, mainly via signaling pathways of epithelial-mesenchymal transition (EMT), cancer stem cell self-renewal, angiogenesis, cell cycle distribution, and apoptosis [12, 27]. Previous studies have explored some possible mechanisms of miR-200 dysregulation in cancer cells at both transcription and epigenetic modification levels. For instance, Bracken CP *et al.* showed that ZEB1 and SIP1 could negatively regulate miR-200b∼200a∼429 transcription in Madin-Darby canine kidney (MDCK) cells and human breast cancer cells by binding paired E-box sites [22]. Emily C. Knouf *et al.* identified p73 and p63 as activators of miR-200 family transcription [28]. S-M Ahn *et al.* concluded that Smad3 could directly bind to a Smad-binding element located in the promoter region of miR-200b/a and function as a transcriptional activator [29].

Considering that a single miRNA is capable of affecting multiple target genes in posttranscriptional level, it is not surprising that miRNAs can be directly or indirectly restrained by their target genes, resulting in the formation of complex regulatory feedback networks [30] [31]. Current findings indicate that these bi-directional circuits exist widely in biological processes such as differentiation and development, cell cycle and apoptosis, EMT and so on. For instance, the regulatory feedback loops such as Notch/miR-326 [32], E2F1/miRNA-223 [33], NF-κB/miR-200b [34], ZEB/miR-200 [22], p53/miR-200 [35] and C/EBPα-miR-34a-E2F3 [36] have been illuminated their molecular networks one after another.

On miR-200b/E2F3 signaling pathway [21], E2F3 is a member of the transcription factor E2F family and has two distinct isoforms, E2F3a and E2F3b, which share DNA binding, heterodimerisation and pocket protein binding domains and differ only in their N-termini [37]. The E2F family includes both transcriptional activators (E2F1, E2F2, and E2F3a) and repressors (E2F3b, E2F4-E2F8). Activator E2Fs are important in the control of cell proliferation by releasing from pRb, and binding to the promoters of target genes via their response elements during late G1/S phase of cell cycle. Repressor E2Fs constrain the transcription of E2F target genes when cooperate with the pocket proteins p107 and p130 and with the addition of repressive histone deacetylases during the early G1 phase [38]. Mounting evidence indicates that E2F3 plays as an oncogene in the regulation of tumorigenesis. For example, Na Lu Smith *et al.* showed that E2F3 was up-regulated as part of a “preneoplastic expression profile” in fallopian tube epithelium of women with BRCA1 mutations. BRCA1 haploinsufficiency in normal fallopian tube epithelium may lead to up-regulation of E2F3b and increased proliferation before the development of intraepithelial neoplasia [39]. Foster CS *et al.* suggested that the pRB-E2F3-EZH2 control axis may have a critical role in modulating aggressiveness of individual human prostate cancer [40]. In urinary bladder cancer, amplification of 6p22 sequences that included E2F3 was concerned with higher cancer cell proliferation rates [41]. Peng *et al.* reported that miR-200 and its targets Sox2 and E2F3 were engaged in an unilateral negative feedback loop to direct cell-cycle exit and differentiation of neural progenitors [42].

Returning to the hypothesis posed at the beginning of our present study, it is now possible to substantiate these novel findings: (1) E2F3b levels are obviously up-regulated in docetaxel-resistant LAD cells compared with parental cells. (2) Overexpression of E2F3b, not E2F3a, could noticeably decline the level of miR-200b, and inhibition of E2F3 significantly increases the level of miR-200b. (3) Down-regulation of E2F3b helps to restore the expression of miR-200b and resensitize docetaxel-resistant LAD cells. (4) Down-regulation of E2F3b suppresses cell proliferation, stimulates cell apoptosis, induces G2/M cell cycle arrest and ultimately reverses both the *in vitro* and *in vivo* chemoresistance of docetaxel-resistant LAD cells in a miR-200b-dependent manner. Based on our previous study, E2F3 was validated as a direct target of miR-200b [21]. Now we further demonstrate that miR-200b expression is negatively regulated by E2F3b, suggesting a double-negative feedback loop between E2F3b and miR-200b, which could be a potential mechanism for reversing chemoresistance of LAD.

It was raised by Blagosklonny MV *et al.* that the cell fate during mitotic arrest was determined by the proclivity to apoptosis and strength of mitotic checkpoint [43–46]. During mitotic arrest caused by microtubule–active drugs such as docetaxel, transcription is inhibited. If a cell can tolerate the decline in anti-apoptotic proteins, then a decrease in cyclin B1 will ensure mitotic slippage and resumed transcription, resulting in a multi-nucleated cell, if not, the cell likely dies via apoptosis. Moreover, decrease of Mdm-2 during transcription inhibition leads to p53 accumulation and induction of p21 and Bax following mitotic slippage, resulting in G1 arrest (senescence) and apoptosis, respectively. According to our results, up-regulation of miR-200b or down-regulation of E2F3b could not only enhance docetaxel-resistant LAD cell apoptosis but also arrest cells at G2/M phase, suggesting a stonger mitotic checkpoint and a lower threshold of apoptosis activation brought by the miR-200b/E2F3 feedback loop.

In conclusion, we describe here a novel mechanism in which E2F3b and miR-200b each suppresses the other, suggesting a novel functional double-negative feedback loop. Artificial controls on this circuit through ectopic miR-200b delivery or E2F3b knockoff may be potential reversal strategies in overcoming lung cancer chemoresistance.

## MATERIALS AND METHODS

### Cell lines and reagents

Human LAD cell lines SPC-A1 and H1299 were acquired from the Tumor Cell Bank of Chinese Academy of Medical Science (Shanghai, China). The docetaxel-resistant SPC-A1 cell line (SPC-A1/DTX) was established as described in our previous work and preserved in a final concentration of 50.0ug/L of docetaxel in our laboratory. The docetaxel-resistant H1299 cell line (H1299/DTX) was also established and preserved in 50.0ug/L docetaxel [21, 47]. SPC-A1, SPC-A1/DTX, H1299, and H1299/DTX cells were all maintained in RPMI 1640 medium containing 10% fetal bovine serum and ampicillin and streptomycin in humidified.

### Plasmid construction and cell transfection of oligonucleotides and plasmids

For E2F3a (GenBank no.NM_001949) gene and E2F3b (GenBank no.NM_001243076) gene expression encoding box, pcDNA vectors were constructed via designing PCR primers accorded to LAD cell line SPC-A1 as cDNA templates using PCR amplification and cloning of eukaryotic expression (purchased from Invitrogen, shanghai). Online design software was applied to design primers and BLAST homology screening was subsequently performed. Primers were synthesized by Sangon Biotech (shanghai) co., ltd. with the sequences as follows: E2F3a sense: 5′-TTTAAACCATCTGAGAGGTACTGATGA-3′, reverse: 5′-CGGCCCTCCGGCAA-3′; E2F3b sense: 5′-TTTAAACCATCTGAGAGGTACTGATGA-3′, reverse: 5′-CCCTTACAGCAGCAGGCAA-3′. All the above sequences were inserted into the BglII and HindIII enzyme sites of pSilencer4.1-CMVneo vector, respectively. The recombinant plasmids were named pcDNA/E2F3a, pcDNAE2F3b, pSil/shE2F3-1∼3 and pSil/shcontrol, respectively. The primers used for pSil/shE2F3-1∼3 and pSil/shcontrol were presented in Supplementary Table 1.

MiR-200b mimics, miR-200b mimics control, miR-200b inhibitor, and miR-200b inhibitor control were all purchased from GenePharma (Shanghai, China). The sequence of miR-200b inhibitor was 5′-UCAUCAUUACCAGGCA GUAUUA-3′. MiR-200b inhibitor/NC sequence was 5′-UUCUCCGAACGUGUCACGUTT-3′. MiR-200b mimics primers were designed as follows: sense: 5′-UAAUACUGCCUGGUAAUGAUGA-3′, reverse: 5′-AUCAUUACCAGGCAGUAUUAUU-3′. MiR-200b mimics/NC sequence was: sense: 5′-UUCUCCGAACGUGUCACGUTT-3′, reverse: 5′-ACGUGACACGUUCGGAGAATT-3′.

Cells were planted into 6-well plates (2×10^5^ cells/well) and transfected with 4ug DNA or 100pmol miRNAs using si-RNA Mate (GenePharma, China) or Turbofect Transfection Reagent (Thermo Scientific, USA) according to the manufacturer's protocol.

### RNA extraction and qRT-PCR analysis

Total RNA was extracted from the cultured cells using Trizol (Invitrogen, CA, USA) according to the manufacturer's protocol and concentration was measured by a spectrophotometer. Reverse transcription was conducted using TaqmanTM microRNA reverse transcription kit and subjected to real-time PCR using TaqManTM MicroRNA Assay kit (Applied Biosystems, USA) based on the manufacturer's instructions. PCR primers were designed as follows: miR-200b sense: 5′-GCGGCTAATACTGCCTGGTAA-3′, reverse: 5′-GTGCAGGGTCCGAGGT-3′. MiRNA and mRNA levels were normalized to U6 r-RNA and GAPDH mRNA, respectively, calculated with the 2-ΔΔCt methods. GAPDH sense: 5′-GGAGTCAACGGATTTGGTCG-3′, reverse: 5′-CATCGCCCCACTTGATTTTG-3′.U6 sense: 5′-CGCTTCGGCAGCACATATACTA-3′, reverse: 5′-CGCTTCACGAATTTGCGTGTCA-3′. The expression levels were relative to the fold change of the corresponding control cells defined as 1.0. PCR conditions were 30-35 cycles consisted of denaturation at 95°C for 15 seconds, annealing at 60°C for 60 seconds, and extension at 60°C for 60 seconds.

### ChIP assay

ChIP assay was performed with Immunoprecipitation Assay Kits (Millipore) according to the manufacturer's instructions. Briefly, cells were cross-linked with 1% formaldehyde for 10 minutes at 37°C. The cells were then resuspended in 200μl of lysis buffer and incubated for 10 minutes on ice. The lysate was sheared to lengths between 200 and 1000 base pairs by sonication. The supernatant was pre-cleared with a Salmon Sperm DNA/ Protein A Agarose-50% Slurry. The recovered supernatant was incubated with antibodies E2F3 (1-2μg per 100-500μg of total protein) (Santa Cruz Biotechnology, USA) or an isotype control IgG (15μg rabbit polyclonal or 5μg mouse monoclonal IgG) (Millipore) overnight at 4°C with rotation. Next, the antibody/DNA complex was collected using Salmon Sperm DNA/Protein A Agarose Slurry for 1 hour at 4°C with rotation, and the complex was eluted by elution buffer. Crosslinks were reversed with 5M NaCl heating at 65°C for 4 hours. The DNA sample was then purified and measured by qRT-PCR.

### Dual luciferase reporter assay

SPC-A1 and SPC-A1/DTX cells were seeded into 96-well plates (4×10^3^/well) and cotransfected with luciferase reporter plasmids with (or without) pcDNA/E2F3b using Roche X-tremeGENE HP DNA Transfection Reagent. The pLUC firefly luciferase vectors contained empty, wild-type, and mutant miR-200b 3′-UTR sequence, respectively. Luciferase activity assays for miRNA target validation were performed 48 hours after transfection, using the Dual-Luciferase Assay kit (Promega, USA). The relative luciferase activities were normalized by Renilla luciferase activities. The data were relative to the fold change of the corresponding control groups defined as 1.0.

### Western blot analysis

Cells were harvested directly or 48-72 hours after transfection. Cell protein lysates were separated in 10% sodium dodecyl sulfate-polyacrylamide gels, electrophoretically transferred to polyvinylidene difluoride membranes (Roche). Protein loading was estimated using mouse anti-GAPDH monoclonal antibody. The membrane was blotted with 5% skim milk, washed and then probed with the rabbit anti-human E2F3 (1: 400 dilution) and GAPDH (1: 5000 dilution), followed by treatment with secondary antibody conjugated to horseradish peroxidase. The proteins were perceived by the enhanced chemiluminescence kit (Invitrogen) and exposed to x-ray film. All antibodies were purchased from Santa Cruz Biotechnology, USA. Protein levels were normalized to GAPDH.

### Chemosensitivity assay *in vitro*

The single-cell suspensions were seeded and dispersed in 96-well plates (2×10^3^ cells/well). Cells were disposed directly or 24 hours after transfection and allowed to attach overnight. Freshly prepared docetaxel was then added with different final concentrations. After incubation for 48 hours with docetaxel, the 3-(4,5-Dimethyl-2- thiazolyl)-2,5-diphenyl-2H-tetrazolium bromide (MTT) (Sigma, USA) solution (0.5 mg/ml) was added. Following incubation for 4 hours, 150μL of extraction buffer was added into each well. After an overnight incubation, absorbance at 490 nm was measured using a microplate reader (Bio-Rad, Model 680).

### Colony formation assay

Cells were cultured to single cell suspensions and seeded into 6-well plates (500 cells/well) for approximately 24 hours under standard conditions. With specific treatments directly or 48 hours after transfection, and after 14 days cultured in RPMI 1640 medium, cells were fixed with methanol and stained with 0.5% crystal violet. The number of visible colonies was manually calculated.

### Flow cytometric analysis of apoptosis

Cells were reaped directly or 48 hours after transfection via ethylene diamine tetraacetic acid–free trypsinization. Annexin V-fluorescein isothiocyanate (FITC) apoptosis detection kit (Oncogene Research Products, Boston, MA) was applied to detect early apoptosis rate based on the manufacturer's instructions.

### Flow cytometric analysis of cell cycle distribution

Cells were collected directly or 48 hours after transfection and washed with ice-cold phosphate-buffered saline (PBS), and fixed with 70% ethanol overnight at -20°C. Fixed cells were rehydrated in PBS for 10 minutes and incubated in RNase A (1mg/ml) for 30min at 37°C, then the cells were subjected to PI/RNase staining followed by flow cytometric analysis using a FACScan instrument (Becton Dickinson, Mountain View, CA) and Cell Quest software (Becton Dickinson, San Jose, CA) as described previously [48].

### Mice xenograft models and immunohistochemistry

BALB/c athymic nude mice (male, SPF, 4-6 weeks) were purchased from the Department of Comparative Medicine in Jinling Hospital (Nanjing, China). All animal experiments has been strictly conducted in accordance with the ethical standards and performed in accordance with institutional guidelines. Approximately 5.0 × 10^6^ SPC-A1 and SPC-A1/DTX cells, 48 hours after transfection with pSil/shE2F3 or pSil/shcontrol (NC), four groups of cells were harvested and suspended in 100uL PBS, then injected subcutaneously into nude mice on the right side of the posterior flank (n=4 mice per group). Tumor growth was examined every other day, and tumor volumes were calculated using the equation V (in mm^3^)=A×B^2^/2, with A being the largest diameter and B being the perpendicular diameter. When the average tumor size reached nearly 50 mm^3^, docetaxel was dispensed via intraperitoneal injection with a concentration of 1 mg/kg, one dose every other day, with 3 doses in total [24]. All mice were sacrificed 35 days after the first administration of docetaxel. Transplanted tumors were excised, and tumor tissues were performed H&E staining. Transplanted tumor tissues were immunostained for E2F3, Ki-67 and PCNA as described previously [49]. Expression was identified as positive when 50% or more of cancer cells were stained.

### TUNEL assay

Apoptosis in transplanted-tumor tissues was scrutinized by terminal deoxynucleotidyl transferase- mediated dUTP nick end labeling TUNEL method [50]. Tumor tissues were fixed with formaldehyde and then permeabilized with ethanol to allow penetration of the TUNEL reaction reagents into the cell nucleus. Following fixation and washing, incorporation of biotinylated-dUTP onto the 3′-ends of fragmented DNA was carried out in a reaction containing terminal deoxynucleotidyl transferase. The TUNEL assay was performed according to the guidelines recommended by the TUNEL assay kit (KeyGen, Nanjing, China).

### Statistical analysis

The SPSS 17.0 software (SPSS Inc., Chicago, IL, USA) was used for statistical analysis. Experimental data were presented as the means ± standard error of at least three independent experiments. Multiple group comparisons were analyzed with one-way analysis of variance (ANOVA) and two group comparisons were assessed by Student's t-test. All tests performed were two-sided. Significance was accepted at *p*<0.05.

## SUPPLEMENTARY TABLE



## Abbreviations

LAD: lung adenocarcinoma
NSCLC: non-small cell lung cancer
miRNA: microRNA
DTX: docetaxel
qRT-PCR: real-time quantitative reverse-transcription polymerase chain reaction
siRNA: small interfering RNA
ChIP: chromatin immunoprecipitation
H&E: hematoxylin and eosin
PCNA: proliferating cell nuclear antigen.
