# Supplementary material for: A double-negative feedback loop between E2F3b and miR- 200b regulates docetaxel chemosensitivity of human lung adenocarcinoma cells
Source: Oncotarget. 2016 Mar 25;7(19):27613–26. doi: 10.18632/oncotarget.8376 (PMC5053675; doi:10.18632/oncotarget.8376)
Supplement: Supplementary file 1 [file oncotarget-07-27613-s001.pdf]

A double-negative feedback loop between E2F3b and miR-200b regulates docetaxel chemosensitivity of human lung adenocarcinoma cells

SUPPLEMENTARY TABLE

Supplementary Table S1: The primers used for pSil/shE2F3-1~3 and pSil/shcontrol

ID	seq
E2F3(pcDNA/E2F3a)-P1	GTCCGGACTCAGATCTCGAGCTATGAGAAAGGGAATCCAGC
E2F3(pcDNA/E2F3a)-P2	TATCTAGATCCGGTGGATCCTCAACTACACATGAAGTCTTCCACC

ID	seq
E2F3(pcDNA/E2F3b)-P1	GTCCGGACTCAGATCTCGAGCTATGCCCTTACA GCAGCAGGCAAAGCGAAGGCTGGAG
E2F3(pcDNA/E2F3b)-P2	TATCTAGATCCGGTGGATCCTCAACT ACACATGAAGTCTTCCACC

ID	5’	stem	loop	stem	3’
E2F3-RNAi(1)-a	GATCCC	ccAACTCAGGA CATAGCGATT	CTCGAG	AATCGCTATGTC CTGAGTTGG	TTTTTGGAT
E2F3-RNAi(1)-b	AGCTATCCAAAAA	ccAACTCAGGA CATAGCGATT	CTCGAG	AATCGCTATGTC CTGAGTTGG	GG
E2F3-RNAi(2)-a	GATCCC	ccTGACTCAATAG AGAGCCTA	CTCGAG	TAGGCTCTCTAT TGAGTCAGG	TTTTTGGAT
E2F3-RNAi(2)-b	AGCTATCCAAAAA	ccTGACTCAATAG AGAGCCTA	CTCGAG	TAGGCTCTCTATT GAGTCAGG	GG
E2F3-RNAi(3)-a	GATCCC	ccAAACTGTTATAG TTGTGAA	CTCGAG	TTCACAACTATAA CAGTTTGG	TTTTTGGAT
E2F3-RNAi(3)-b	AGCTATCCAAAAA	ccAAACTGTTATA GTTGTGAA	CTCGAG	TTCACAACTATA ACAGTTTGG	GG
